# Nivolumab therapy for metastatic collecting duct carcinoma after nephrectomy: A case report

**DOI:** 10.1097/MD.0000000000013173

**Published:** 2018-11-09

**Authors:** Shotaro Yasuoka, Tsutomu Hamasaki, Eigo Kuribayashi, Masato Nagasawa, Takanori Kawaguchi, Yoji Nagashima, Yukihiro Kondo

**Affiliations:** a Department of Urology; b Department of Pathology, Aidu Chuo Hospital, Tsurugacho, Aiduwakamatu-shi, Fukushima; c Department of Surgical Pathology, Tokyo Women‘s Medical University Hospital, 8-1 Kawada-cho, Shinjuku-ku; d Department of Urology, Nippon Medical School Hospital, Sendagi, Bunkyo-ku, Tokyo, Japan.

**Keywords:** collecting duct carcinoma, nivolumab, PD-1 immune checkpoint inhibitor antibody, PD-L1, performance status

## Abstract

**Rationale:**

Collecting duct carcinoma (CDC) is a rare type of nonclear renal cell carcinoma, often presenting at an advanced stage of the disease, and standard treatment guidelines have not been established.

**Patient concerns:**

A 73-year-old man was admitted to our hospital with complaints of fever and lower right back pain.

**Diagnoses:**

Computed tomography revealed a poorly defined tumor of the right kidney without metastasis. The patient underwent right radical nephrectomy and was diagnosed with clinical stage T1bN0M0 renal cancer; the pathological findings showed collecting duct carcinoma.

**Interventions:**

After nephrectomy, multiple lung metastases were found in the following month, so first-line chemotherapy of gemcitabine (1000 mg/m^2^ on days 1 and 8, every 21 days) and cisplatin (70 mg/m^2^ on day 2, every 21 days) was administered. Due to disease progression, targeted therapy with axitinib (10 mg/body) and second-line chemotherapy of paclitaxel (200 mg/m^2^ on day 1, every 21 days) and carboplatin (area under the curve of 6 on day 1, every 21 days) were subsequently administered. However, the lung metastases progressed and new metastases spread to the right adrenal gland, liver, and lymph nodes. Based on the high expression of programmed death-ligand 1 in tumor cells, we treated the patient with the immune checkpoint inhibitor nivolumab.

**Outcomes:**

After 2 courses of treatment, he experienced a partial response and improved performance status, and thus was discharged from the hospital. To date, the patient is on his fifth course of treatment as an outpatient without disease progression.

**Lessons:**

The findings of our study suggest that nivolumab may be effective even if the patient has highly progressive CDC with a low PS, if PD-L1 is highly expressed in the tumor cells.

## 1 Introduction

Collecting duct carcinoma (CDC) is a rare type of nonclear renal cell carcinoma, often presenting at an advanced stage of the disease. Up to 40% of patients have metastatic spread at initial presentation, and most die within 1 to 3 years from diagnosis.^[1]^ CDC originates in the distal collecting duct and shares biologic features with urothelial carcinoma.^[2,3]^ Surgical treatment of the primary lesion is performed in many cases, after which immunotherapy, chemotherapy, and targeted therapy are performed as systemic treatments.^[4,5]^ Because CDC is rare, it has been difficult to conduct large-scale clinical trials, and standard treatment guidelines have not been established.

Immune checkpoint inhibitors are currently used to treat renal cell carcinoma and urothelial carcinoma. Nivolumab is a programmed cell death protein 1 (PD-1) immune checkpoint inhibitor antibody that selectively blocks the interaction between PD-1, which is expressed on activated T cells, and programmed death-ligand 1 (PD-L1) and PD-L2, which are expressed on immune cells and tumor cells.^[6]^ This inhibition of binding between PD-1 and its ligands by nivolumab stimulates the apoptosis of activated T cells, resulting in antitumor effects.

In this study, we describe a case of rapidly advanced recurrence of CDC after nephrectomy that markedly responded to immune checkpoint inhibitor therapy with nivolumab.

## 2 Case report

**Figure 1:**
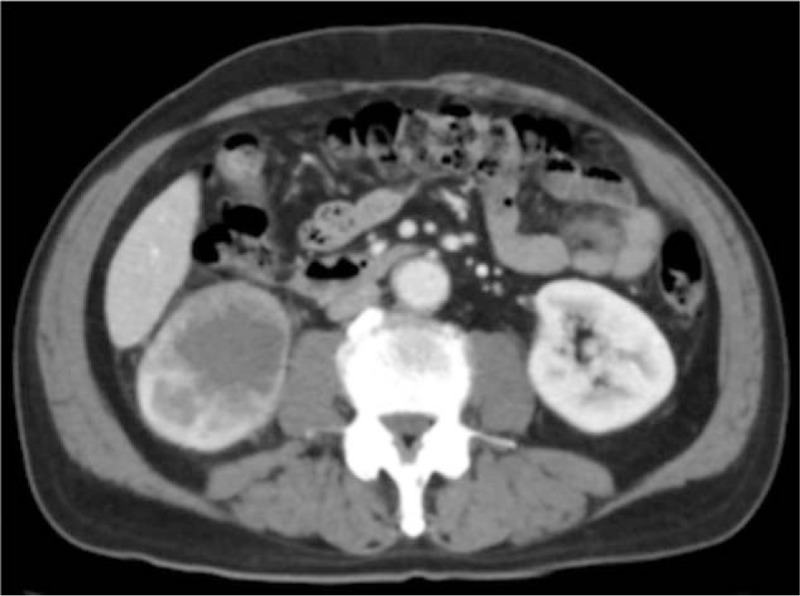
Contrast-enhanced abdominal CT revealed a poorly enhanced tumor of 55 mm in diameter in the inferior pole of the right kidney. CT = computed tomography.

**Figure 2:**
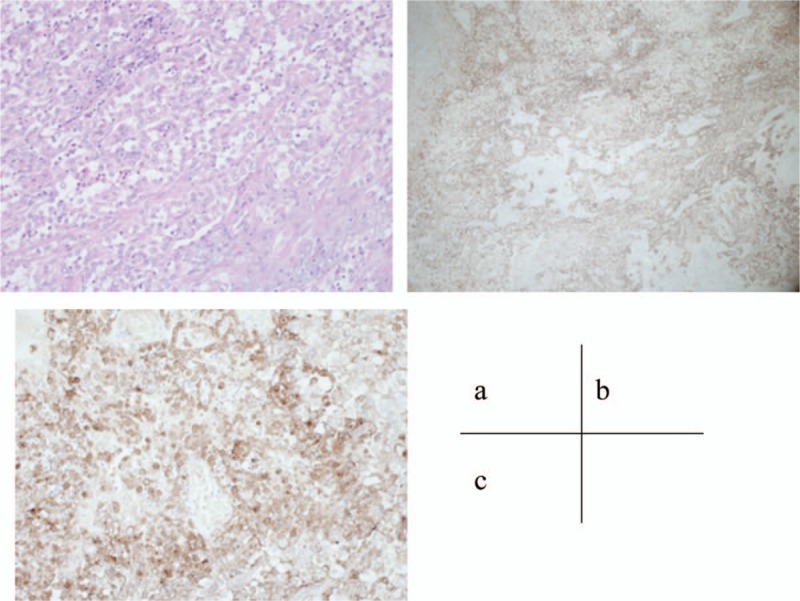
High-power view of hematoxylin and eosin (HE)-stained sections [(A) HE, 200×] and low-power and high-power view of PD-L1 in surgical specimens [(B) PD-L1, 40×; (C) PD-L1, 200×]. PD-L1 was highly expressed in renal tumor cells. HE = hematoxylin and eosin.

This case report was approved by the research ethics committee of Aidu Chuo Hospital. A 73-year-old man was admitted to our hospital with complaints of fever and lower right back pain. He had a past history of nonmuscle invasive bladder cancer, which had been treated with transurethral resection of the bladder tumor and intravesical immunotherapy with Bacillus Calmette-Guerin. His Eastern Cooperative Oncology Group performance status (PS) was 0. Initial laboratory findings revealed a white blood cell count of 13560/μL and a C-reactive protein level of 8.14 mg/dL. Computed tomography (CT) showed a poorly defined tumor of 55 mm in diameter in the inferior pole of the right kidney (Fig. 1). No obvious metastasis was observed. Because renal abscess was suspected, ultrasound-guided biopsy of the lesion was performed. The majority of biopsy specimens were necrotic tissue, and structural destruction and abnormal cell proliferation were observed; thus, malignancy could not be denied. Radical nephrectomy of the right kidney was performed, and histopathological diagnosis showed CDC with Fuhrman grade 4. The expression of PD-L1 was examined in the primary lesion and high expression was found in the tumor cells (Fig. 2). The patient complained of being in poor physical condition 1 month after surgery, and CT scans showed multiple lung metastases. Thus, six courses of chemotherapy with a combination of gemcitabine (1000 mg/m^2^ on days 1 and 8, every 21 days) and cisplatin (70 mg/m^2^ on day 2, every 21 days) were administered as first-line treatment, after which CT scans showed a partial response (PR). However, 1 month after completing chemotherapy, CT scans revealed progression of the lung lesions; thus, targeted therapy was performed with 10 mg axitinib for 1 month. Because the lung metastases progressed and the patient's PS worsened to 1, the treatment was changed to a combination of paclitaxel (200 mg/m^2^ on day 1, every 21 days) and carboplatin (area under the curve of 6 on day 1, every 21 days) as second-line chemotherapy for 2 months. However, the lung metastases progressed and spread to the right adrenal gland, liver, and lymph nodes, and his PS worsened to 2, requiring the patient to be hospitalized.

**Figure 3:**
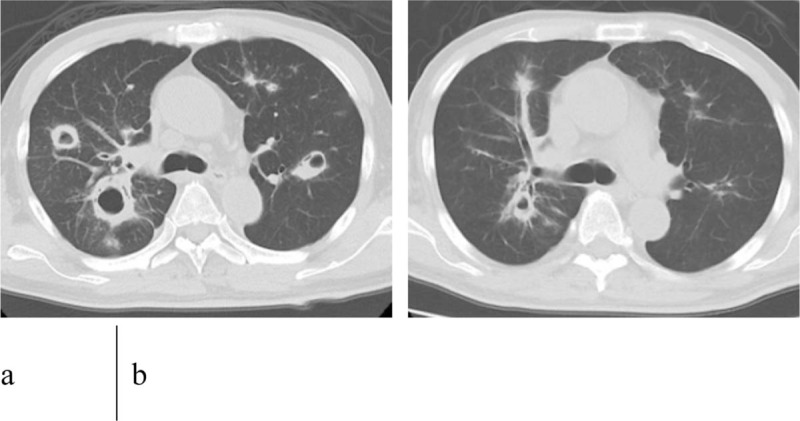
Chest CT shows changes in lung metastases. Just before initiating nivolumab treatment (A), and after 2 months of nivolumab treatment (B). CT = computed tomography.

Based on the high expression of PD-L1 in tumor cells, the patient received immune checkpoint inhibitor therapy with nivolumab (3 mg/kg on day 1, every 14 days) as an inpatient. After completion of the first course of treatment, his PS remained unchanged, and a chest x-ray showed that the lung metastases had increased in size. However, during the second course of treatment, the patient's general condition began to gradually improve each day. Furthermore, after completing 2 courses of nivolumab, CT scans revealed a PR of lung metastases and other lesions also shrank (Fig. 3). The patient's PS improved from 2 to 1, allowing him to be discharged from the hospital. To date, the patient is on his fifth course of treatment as an outpatient, and has not experienced adverse effects or disease progression.

## 3 Discussion

The only prospective study on the treatment of CDC was reported by Oudard et al^[3]^ According to that regimen of gemcitabine plus cisplatin or carboplatin, an objective response rate of 26% was induced in a patient with metastatic CDC.^[3]^ In many cases, patients with metastatic CDC are treated based on the treatment strategy for urothelial carcinoma.^[5]^ Because this therapy is considered the standard treatment for metastatic CDC, we used it as first-line chemotherapy. The effects of targeted therapy on the treatment of CDC have not yet been established.^[5]^

To the best of our knowledge, there have only been 2 case reports that have described the efficacy of immune checkpoint inhibitors against CDC. Rimar et al^[7]^ reported the first case in which nivolumab was used in a patient with CDC, who had initially been treated with chemotherapy and subsequently treated with a tyrosine kinase inhibitor; this patient achieved PR with nivolumab therapy.^[7]^ Mizutani et al^[8]^ also reported a patient with metastatic CDC who responded well to nivolumab therapy. Moreover, the surgical specimens from this patient highly expressed PD-L1. Both reports demonstrated the possibility that nivolumab could be effective in treating CDC, and our case report supports their findings.

In immune checkpoint inhibitor therapy, a phenomenon in which the lesions temporarily worsen has been reported as pseudo progression.^[9]^ Therefore, evaluation criteria other than the conventional World Health Organization evaluation and Response Evaluation Criteria in Solid Tumors have been established.^[10]^ Even in this case study, although the lung metastases temporarily progressed during the first course of nivolumab therapy, it was not certain whether progression of the disease or pseudo progression occurred. Nevertheless, we confirmed that a PR was achieved after 1 month of immune checkpoint inhibitor therapy, because the patient's PS clearly improved and his lung and other metastases clearly shrank after the second course of treatment.

The high expression of PD-L1 in clear cell renal carcinoma and non-clear cell renal carcinoma correlates with a poor prognosis.^[11,12]^ However, the correlation between PD-L1 expression and the response rate to nivolumab is unclear.^[13]^ Mizutani et al^[8]^ reported that high PD-L1 expression in CDC contributes to a favorable clinical response to nivolumab, and the findings from our case study are in accordance with that study. It can be presumed that the high expression of PD-L1 found in our case was a prognostic indicator of the high malignant potential of the carcinoma and the patient's poor clinical outcome. His PS worsened as the disease progressed, requiring him to be hospitalized. However, the patient's general condition markedly improved with administration of nivolumab, which allowed him to receive therapy as an outpatient.

The findings of our study suggest that nivolumab may be effective even if the patient has highly progressive CDC with a low PS, if PD-L1 is highly expressed in the tumor cells. A large-scale prospective study of patients with CDC treated with nivolumab is needed to confirm our results.

## Author contributions

**Supervision:** Masato Nagasawa, Takanori Kawaguchi.

**Writing – original draft:** Shotaro Yasuoka.

**Writing – review & editing:** Tsutomu Hamasaki, Eigo Kuribayashi, Yoji Nagashima, Yukihiro Kondo.

## References

[1] National Comprehensive Cancer Network (NCCN) Clinical Practice Guidelines in Oncology for Kidney Cancer. Version 2.2016. 10.6004/jnccn.2016.0051 27059193

[2] Chao D Zisman A Pantuck AJ Collecting duct renal cell carcinoma: clinical study of a rare tumor. J Urol 2002;167:71–4. 11743278 10.1016/s0022-5347(05)65385-2

[3] Oudard S Banu E Vieillefond A Prospective multicenter phase II study of gemcitabine plus platinum salt for metastatic collecting duct carcinoma: results of a GETUG (Groupe d‘Etudes des Tumeurs Uro-Genitales) study. J Urol 2007;177:1698–702. 17437788 10.1016/j.juro.2007.01.063

[4] Tokuda N Naito S Matsuzaki O Collecting duct (Bellini duct) renal cell carcinoma: a nationwide survey in Japan. J Urol 2006;176:40–3. 16753362 10.1016/S0022-5347(06)00502-7

[5] Dason S Allard C Sheridan-Jonah A Management of renal collecting duct carcinoma: a systematic review and the McMaster experience. Curr Oncol 2013;20:e223–32. 23737692 10.3747/co.20.1230 PMC3671029

[6] Motzer RJ Escudier B McDermott DF Nivolumab versus everolimus in advanced renal-cell carcinoma. N Engl J Med 2015;373:1803–13. 26406148 10.1056/NEJMoa1510665 PMC5719487

[7] Rimar KJ Meeks JJ Kuzel TM Anti-programmed death receptor 1 blockade induces clinical response in a patient with metastatic collecting duct carcinoma. Clin Genitourin Cancer 2016;14:e431–4. 27021586 10.1016/j.clgc.2016.02.013

[8] Mizutani K Horie K Nagai S Response to nivolumab in metastatic collecting duct carcinoma expressing PD-L1: a case report. Mol Clin Oncol 2017;7:988–90. 29285361 10.3892/mco.2017.1449 PMC5740828

[9] Chiou VL Burotto M Pseudoprogression and immune-rerated response in solid tumors. J Clin Oncol 2015;33:3541–3. 26261262 10.1200/JCO.2015.61.6870 PMC4622096

[10] Tazdait M Mezquita L Lahmar J Patterns of responses in metastatic NSCLC during PD-1 or PDL-1 inhibitor therapy: Comparison of RECIST 1.1, irRECIST and iRECIST criteria. Eur J Cancer 2018;88:38–47. 29182990 10.1016/j.ejca.2017.10.017

[11] Xu F Xu L Wang Q Clinicopathological and prognostic value of programmed death ligand-1(PD-L1) in renal cell carcinoma: a meta-analysis. Int J Clin Exp Med 2015;8:14595–603. 26628942 PMC4658831

[12] Choueiri TK Fay AP Gray KP PD-L1 expression in nonclear-cell renal cell carcinoma. Ann Oncol 2014;25:2178–84. 25193987 10.1093/annonc/mdu445 PMC4288138

[13] Zhang T Xie J Arai S The efficacy and safety of anti-PD-1/PD-L1 antibodies for treatment of advanced or refractory cancers: a meta-analysis. Oncotarget 2016;7:73068–79. 27683031 10.18632/oncotarget.12230 PMC5341964

